# Antisense RNA C9orf72 hexanucleotide repeat associated with amyotrophic lateral sclerosis and frontotemporal dementia forms a triplex-like structure and binds small synthetic ligand

**DOI:** 10.1093/nar/gkae376

**Published:** 2024-05-13

**Authors:** Leszek Błaszczyk, Marcin Ryczek, Bimolendu Das, Martyna Mateja-Pluta, Magdalena Bejger, Joanna Śliwiak, Kazuhiko Nakatani, Agnieszka Kiliszek

**Affiliations:** Institute of Bioorganic Chemistry, Polish Academy of Sciences, Z. Noskowskiego 12/14, 61-704, Poland; Institute of Bioorganic Chemistry, Polish Academy of Sciences, Z. Noskowskiego 12/14, 61-704, Poland; Department of Regulatory Bioorganic Chemistry, SANKEN (The Institute of Scientific and Industrial Research), Osaka University, 8-1 Mihogaoka, Ibaraki 567-0047, Japan; Institute of Bioorganic Chemistry, Polish Academy of Sciences, Z. Noskowskiego 12/14, 61-704, Poland; Institute of Bioorganic Chemistry, Polish Academy of Sciences, Z. Noskowskiego 12/14, 61-704, Poland; Institute of Bioorganic Chemistry, Polish Academy of Sciences, Z. Noskowskiego 12/14, 61-704, Poland; Department of Regulatory Bioorganic Chemistry, SANKEN (The Institute of Scientific and Industrial Research), Osaka University, 8-1 Mihogaoka, Ibaraki 567-0047, Japan; Institute of Bioorganic Chemistry, Polish Academy of Sciences, Z. Noskowskiego 12/14, 61-704, Poland

## Abstract

The abnormal expansion of GGGGCC/GGCCCC hexanucleotide repeats (HR) in *C9orf72* is associated with amyotrophic lateral sclerosis (ALS) and frontotemporal dementia (FTD). Structural polymorphisms of HR result in the multifactorial pathomechanism of ALS/FTD. Consequently, many ongoing studies are focused at developing therapies targeting pathogenic HR RNA. One of them involves small molecules blocking sequestration of important proteins, preventing formation of toxic nuclear foci. However, rational design of potential therapeutics is hindered by limited number of structural studies of RNA-ligand complexes. We determined the crystal structure of antisense HR RNA in complex with ANP77 ligand (1.1 Å resolution) and in the free form (0.92 and 1.5 Å resolution). HR RNA folds into a triplex structure composed of four RNA chains. ANP77 interacted with two neighboring single-stranded cytosines to form pseudo-canonical base pairs by adopting sandwich-like conformation and adjusting the position of its naphthyridine units to the helical twist of the RNA. In the unliganded structure, the cytosines formed a peculiar triplex i-motif, assembled by trans C•C^+^ pair and a third cytosine located at the Hoogsteen edge of the C•C^+^ pair. These results extend our knowledge of the structural polymorphisms of HR and can be used for rational design of small molecules targeting disease-related RNAs.

## Graphical Abstract

**Graphical Abstract:**
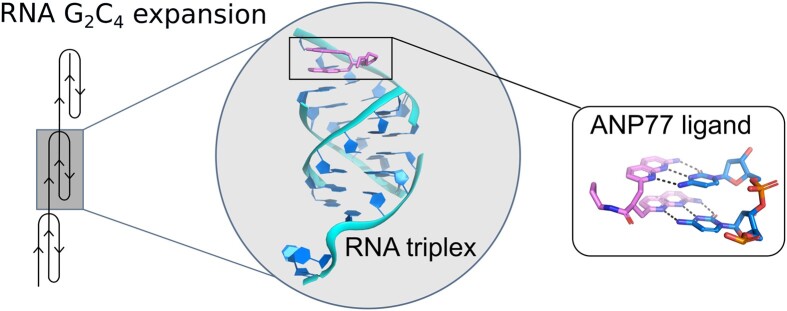

## Introduction

Amyotrophic Lateral Sclerosis and Frontotemporal Dementia are fatal neurodegenerative repeat expansion disorders that affect the motor neurons in the brain and spinal cord. The progressive degeneration of nerve cells leads to changes in behavior, dysphagia, dysarthria, respiratory failure, and consequently, death (1,2). Currently, only symptomatic treatments are available to alleviate disease progression. The most frequent cause of ALS/FTD is a mutation in the *C9orf72* gene, which encodes a protein involved in the autophagy-lysosome pathway (3). The promoter region of *C9orf72* carries microsatellite sequences consisting of hexanucleotide repeats (HR) 5′-GGGGCC-3′/5′-GGCCCC-3′, which can undergo abnormal expansion. Healthy individuals possess up to 20 repeats, whereas the mutated form of the gene contains several thousand HR units (3–5). The presence of expanded repeats has a negative effect on DNA and RNA functions, resulting in the complex pathomechanism of ALS/FTD. One of these pathways involves transcriptional gene silencing and activation of the DNA damage response through the formation of DNA–RNA hybrids (R-loops) (3,6). On the RNA level, bidirectional transcription produces sense and antisense transcripts containing complementary regions of repeated stretches of GGGGCC (G_4_C_2_) and GGCCCC (G_2_C_4_) that form stable secondary and tertiary structures (6,7). As a consequence, mutated transcripts gain the ability to sequester important proteins and form RNA foci that accumulate in the nucleus (8–10). Expanded repeats can also trigger repeat-associated non-ATG (RAN) translation, resulting in toxic cellular polydipeptide aggregates (11–13).

The structural evaluation of the HR repeats has been performed only *in vitro* (in solution). These experiments show that sense and antisense transcripts containing HR fold into tertiary motifs and exhibit structural polymorphisms. Sense G_4_C_2_ RNA is rich in guanine residues. Thus, in the presence of potassium ions, inter- or intramolecular G-quadraplexes can be formed and assembled into G-wire or gel-like phases (6,7,14–17). G_4_C_2_ RNA repeats also fold into hairpin structures, which dominate in the absence of K^+^ ions or at low refolding temperature (6,7,17). The antisense G_2_C_4_ repeats are cytosine rich and can fold into hairpin structures with long helical stems. Alternatively, cytosines can undergo protonation to form i-motifs or triplexes (6,7,18). So far, only one crystal structure of a G_2_C_4_ repeat has been reported (19). It folds into a duplex, representing part of the hairpin stem, consisting of G-C pairs interposed by non-canonical C–C pairs. The 3D structures of G_2_C_4_ repeats with protonated cytosine residues are currently unknown.

The structural polymorphism of the expanded HR has implications for the development of drugs against ALS/FTD. One proposed therapy involves small molecules that can interact with expanded HR and block its protein-binding sites, preventing the formation of toxic foci and inhibiting RAN translation (17). Most investigations have focused on targeting G_4_C_2_ repeats with small molecules using library screening, whereas the druggability of G_2_C_4_ repeats has not been explored (17,20–23). To be effective, targeted therapies require the development of ligands exhibiting high structural specificity against target RNA (23,24). The rational design of lead compounds and potential therapeutics can be greatly improved by three-dimensional structures of RNA in a ligand-bound form. However, this knowledge is limited with regards to disease-related repeat sequences (24,25).

**Figure 1.**
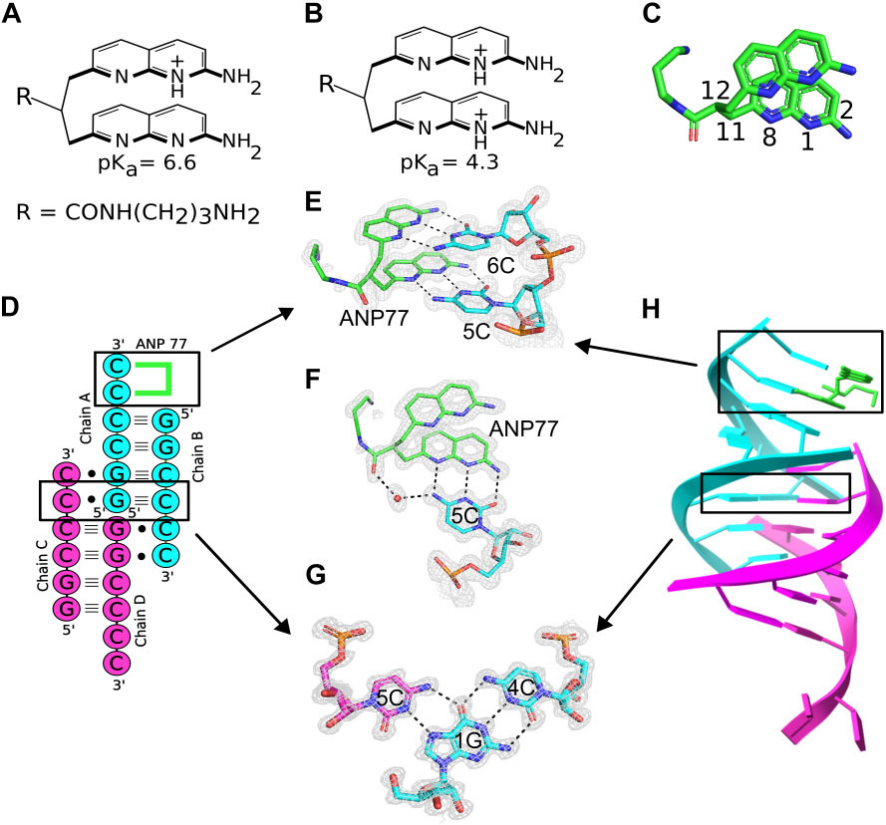
Interactions of ANP77 with G_2_C_4_ RNA. (A, B) chemical structure of the ANP77 molecule: (**A**) in single and (**B**) double-protonated state. (**C**) Stacked conformation of ANP77 observed in the crystal structure. Selected atoms are indicated (see text for details). (**D**) secondary structure of G_2_C_4_–ANP77 complex. Green lines represent ANP77 ligand. (**E, F**) Pseudo-canonical base-pairs formed between ANP77 (green) and cytosine residues from chain A (cyan). **(G)** Base triple between cytosine from chain C (purple), guanosine from chain A (cyan), and cytosine from chain B (cyan). **(H)** Crystal model of RNA tetramer (chain A and B in cyan; chain C and D in pink) with bound ligand molecule (green sticks). The 2Fo–Fc electron density map (gray) is contoured at the 1σ level. The H-bonds are represented by black dashed lines.

Recently, we developed a small molecule ANP77 (2-Amino-1,8-naphthyridine dimer), which belongs to the family of mismatch-binding ligands (MBLs) (26). The MBL molecules were designed to recognize bulges, internal loops or mismatches present in RNA and DNA structures (27–31). They contain at least two heterocyclic moieties that can form hydrogen bonds with nucleobases of single-stranded residues (32,33). The aromatic components are connected by linker that can vary in length and atomic composition, affecting the conformational flexibility of the ligand (26,31). The ANP77 molecule is composed of two 2-amino-1,8-naphthyridine (NU) units connected by a short linker composed of three carbon atoms (Figure 1A). The 2-amino derivatives of the 1,8-naphthyridine were used to bind Watson-Crick interface of cytosines while the linker was restricting conformational diversity and promoting stacked structure of the molecule (26,34,35). Further research using systematic evolution of ligands by exponential enrichment (SELEX) and UV-melting confirmed that ANP77 recognized two consecutive cytosine residues located in single stranded parts of nucleic acids such as bulges or apical loops (26,36,37). Finally, ANP77 was found to interact with cytosine rich sequence of G_2_C_4_ repeats (36).

Here, we present the crystal structure of G_2_C_4_ RNA in complex with the synthetic molecule ANP77. RNA folds into a triplex-like structure involving protonated cytosine residues, whereas ANP77 interacts directly with RNA *via* pseudo-canonical base pairs. These crystallographic models are rare examples demonstrating unexplored potential of cytosine-rich sequences to form complex RNA structures and provide sophisticated crystallographic templates for structure-guided approaches in the development of lead compounds for drug discovery against ALS/FTD.

## Materials and methods

### Synthesis, purification and crystallization of RNA oligomers and ligand

The RNA oligomers were synthesized by the solid phase method using Applied Biosystems DNA/RNA synthesizer and TOM protected phosphoramidites. The synthesis was carried out in DMT-ON mode and RNA was purified according to the protocol suitable for Glen-Pak cartridges (Glen Research). The purified RNA was lyophilized under vacuum using a Speed-Vac and stored at −20°C. The ANP77 was chemically synthesized according to the protocol described before (26). The lyophilized ligand was dissolved in water to the final concentration of 30 mM and used for further research. Before crystallization the RNA was dissolved in buffer containing 10 mM sodium cacodylate pH 7.0 and 100 mM NaCl. The final concentration of RNA was 0.5 mM. The oligomer was denatured for 5 min at 95°C and snap-cooled on ice for 10 min following incubation for 20 min in RT with or without 0.75 mM ANP77. A minimum amount of ligand was added to saturate potential binding sites in the RNA since higher concentration of ligand caused RNA precipitation. Crystals were grown by the sitting drop method at 19°C. The initial screening (covering pH range 5.6–8.5) was performed to determine optimal crystallization conditions. The best hit was observed for buffers with pH 6.0. We have performed crystallization of RNA with and without ANP77 also in other pH values (pH 5.5 and pH 7.0) but usually the sample was precipitating or giving small micro crystals not suitable for X-ray measurements. The crystals of RNA-ligand complex grew in 80 mM NaCl, 40 mM sodium cacodylate trihydrate pH 6.0, 45% v/v MPD, 12 mM spermine tetrahydrochloride. Unliganded RNA oligomer was crystallized in 80 mM NaCl, 20 mM magnesium chloride hexahydrate, 40 mM sodium cacodylate trihydrate pH 6.0, 35% v/v MPD and 12 mM spermine tetrahydrochloride. The crystals of unliganded RNA were also obtained in 0.08M magnesium acetate tetrahydrate, 0.05M sodium cacodylate trihydrate pH 6.5, 30% w/v polyethylene glycol 4000. The crystallization drop contained 2:1 or 1:1 RNA-to-crystallization solution ratio. Crystals appeared within several weeks. The size of the crystals was approx. 0.05 mm with cuboid shape.

### X-ray data collection, structure solution and refinement

X-ray diffraction data were collected on in house diffractometer (XtaLab Synergy-R, Rigaku) and on P13 beam line of the EMBL Hamburg at the PETRA III storage ring (DESY, Hamburg, Germany) (38). The data were integrated using CrysAlisPro software (Rigaku). The data were scaled using SCALA from CCP4 program suite (39,40). The structure of RNA-ligand complex was solved by molecular replacement with PHASER using part of RNA model containing C_4_G_2_ repeats (PDB code: 5ew7) (41). The phases of unliganded RNA model were taken from RNA-ligand structure. Early stages of the refinement were done using the Refmac5 from the CCP4 program suite. Further refinement was carried out with PHENIX (42,43). The manual model building was done using Coot (44). Restraints for the ANP77 ligand were generated by the grade web server (Global Phasing, http://grade.globalphasing.org). All figures were drawn using PyMOL (https://pymol.org). Atomic coordinates of the crystallographic models have been deposited in the Protein Data Bank (accession codes: 8QMH, 8QMI and 9EN6). The statistics of X-ray data collection and refinement are summarized in Supplementary Table S1. X-ray diffraction images are deposited in the MX-RDR database (Macromolecular Xtallography Raw Data Repository) (https://mxrdr.icm.edu.pl/) (doi:10.18150/AJNJBE for RNA-ANP77 structure and doi:10.18150/FESTPM, doi:10.18150/FRXAN8 for native RNA models).

### CSI-TOF-MS measurements

Time-of-flight mass spectrometry analysis by cold spray ionization (CSI-TOF MS) data were obtained in negative mode using JEOL JMS-T100LP AccuTOF LC-plus 4G mass spectrometer. Samples containing the G2C4 RNA (20 μM) with or without ANP77 (50 μM) in an 8:2 mixture of water and methanol containing ammonium acetate (250 mM) were sprayed at a flow rate of 14 μl min^−1^. During the injection, the spray temperature was set at −10°C.

### Circular dichroism measurements

Circular dichroism (CD) experiments were carried out on a J-725 CD spectropolarimeter (JASCO) using a 10 mm path length cell at room temperature. Prior to measurements RNA (2.5 μM) was refolded in sodium cacodylate buffer (10 mM, pH 7.0) containing 100 mM NaCl for 5 min at 95°C and snap-cooled on ice for 10 min following incubation for 20 min at room temperature in the presence or absence of ANP77 ligand (25 μM). For each sample, five spectral scans were accumulated in the range from 220–400 nm.

CD in different temperature and pH values was carried out on a Jasco J-815 spectropolarimeter (JASCO) using a 5 mm path length. The pH-dependent CD spectra of RNA G_2_C_4_ (30 μM), (G_2_C_4_)_2_ (14,6 μM) and (G_2_C_4_)_3_ (10 μM) (refolded in the same way as above) were measured in a buffer containing 40 mM sodium cacodylate pH 5.5–7.0 or Tris–HCl pH 7.5–8.0 and 100 mM NaCl. The temperature-dependent CD spectra of G_2_C_4_ oligomer (30 μM) were obtained in sodium cacodylate buffer (40 mM, pH 6.0) containing 10 mM NaCl at 20–75°C. For each sample, five spectral scans were accumulated in the range from 205 to 340 nm.

### DSC measurements

The RNA oligomers were dissolved in 100 mM sodium chloride and 10 mM sodium cacodylate buffer adjusted to pH 7.0, 6.0 or 5.3. The final concentration of RNA was 300 μM. Samples were dialyzed against buffer overnight at 4°C to equilibrate RNA and the reference solutions. Before DSC measurements, RNA samples were denatured at 95°C for 5 min and snap-cooled on ice for 10 min. Next, the RNA was renatured for 20 min in RT with or without 300 μM ANP77 ligand. DSC experiments were performed on a MicroCal PEAQ-DSC calorimeter (Malvern Instruments Ltd) Each measurement was carried out in five cycles of heating and cooling in the range of 2–110°C and a scan rate of 1°C/min. First, reference scans of the buffer were performed to establish the instrument thermal history and to reach a near perfect baseline repeatability. The results were analyzed using dedicated software implemented by Malvern Instruments. The melting temperature (*T*_m_) was calculated by applying a two-state model fitting.

### ITC measurements

ITC (Isothermal Titration Calorimetry) measurements were performed with a PEAQ-ITC calorimeter (Malvern) at 25°C in 10 mM sodium cacodylate pH 6.0 and 100 mM NaCl. 50 μM RNA: G_2_C_4_, (G_2_C_4_)_2_ and (G_2_C_4_)_3_ was titrated with 500 μM ANP77 ligand until the saturation was observed. G_2_C_4_ RNA was titrated with 19 injections of 2 μl volume, whereas (G_2_C_4_)_2_ RNA with 38 injections separated with 160 s intervals. Data analysis was performed using Origin 7.0 software. The binding parameters were obtained by fitting the binding isotherms to a ‘One set of sites’ model for G_2_C_4_ RNA and ‘Sequential binding sites’ model (assuming two binding sites) for (G_2_C_4_)_2_ RNA. The data obtained for (G_2_C_4_)_3_ RNA could not be fitted and were unrepeatable. All measurements were performed in duplicate.

### Native PAGE

For monitoring migration of RNA oligomers in native conditions 500–1000 pmol of G_2_C_4_, (G_2_C_4_)_2_, (G_2_C_4_)_3_, 6U (6 uridines), 12U (12 uridines) and 18U (18 uridines) was renatured for 5 min at 95°C and slowly cooled (0.1°C/s) to 4°C in buffer containing 40 mM sodium cacodylate pH 6.0 and 100 mM NaCl. Next, glycerol was added to the final concentration of 1%. Samples were analyzed by native gel electrophoresis using 15% gel (19:1 acrylamide/bisacrylamide ratio) in 0.5× TB at 4°C (DNApointer, Biovectis). RNA was visualized by UV shadowing.

## Results and discussion

### The overall structure of the G_2_C_4_–ANP77 complex

The ANP77 ligand was designed to selectively bind to the internal loop of the Y/CC motif in RNA (Y denotes pyrimidine) (36,37). The ligand is composed of two 2-amino-1,8-naphthyridine units connected by a short aliphatic linker having attached aminopropyl carboxamide side chain to increase water solubility and interactions with negatively charged nucleic acids (Figure 1A–C). Each 2-amino naphthyridine unit served as a hydrogen bonding partner for interactions with cytosine residues. At a weakly acidic or neutral pH, protonation occurred at one of the NUs, whereas protonation of the second unit required a lower pH (Figure 1A and B) (36). The preliminary experiments using UV-melting method confirmed the binding of ANP77 to the G_2_C_4_ sequence embedded in duplex structure (data not shown). Based on that finding we performed co-crystallization of G_2_C_4_ RNA with ligand molecule.

The three-dimensional model of the G_2_C_4_–ANP77 complex exhibited a complicated triplex-like fold structure composed of four chains of G_2_C_4_ RNA (Protein Data Bank code 8QMH) (Supplementary Table S1). Chains A and B assembled into duplex AB, whereas chains C and D assembled into duplex CD (Figure 1D). In each duplex, four canonical G–C pairs were present as well as two single-stranded, overhanging cytosines at each 3′ end (Figure 1D). In the crystal lattice, duplexes AB and CD interacted with each other by docking 3′ overhanging cytosines from one end of the duplex into each other's major grooves (Figure 1D). Cytosine from chain B interacted with duplex CD, whereas cytosine from chain C interacted with duplex AB. Consequently, chains A and D were arranged head-to-head, forming a pseudo-continuous strand. Overall, the RNA folded into a parallel triplex-like motif flanked by double-stranded duplexes and terminated by single-stranded cytosines (Figure 1D and H). The triplex motif was composed of four C^+^•G-C pairs representing standard major groove base triples (C^+^ denotes protonated cytosine) (45,46). G and C formed canonical Watson–Crick pairs, whereas C^+^ was located in the major groove and interacted with the Hoogsteen edge of guanosine from the G–C pair. The C^+^•G interaction included two H-bonds: one between the exo-amino group of C and the O6 carbonyl of G, and the second bond between the N3 amino group of C and the N7 imino atom of G (Figure 1G). The latter interaction required the protonation of cytosine, resulting in the conversion of the N3 imino atom of C^+^ to an amino function. Although the crystal model was obtained at atomic resolution, the precise position of the hydrogen atoms could not be assigned. Since hydrogen atoms have a small contribution in scattering of X-rays they can be localized only in high quality and ultra-high resolution structures (47). On the other hand, the chemical knowledge and the distance (2.8–2.9 Å) between nitrogen atoms of cytosines and guanosines in the crystal model clearly indicated the presence of H-bond interactions and protonation of C residues in the observed triples.

In previous studies, G_2_C_4_ RNA was crystallized as a duplex (19). The cytosines were engaged in canonical pairing with guanosines and in the formation of non-canonical C-C pairs. Although cytosines can interact with each other to form H-bonds, crystallographic studies of C-rich sequences, such as CCG or CCUG repeats, have indicated that the system tends to minimize the number of C-C pairs while simultaneously maximizing the number of G-C pairs (19,48,49). This was achieved by strand slippage, resulting in 5′ or 3′ overhanging residues or the formation of other types of non-canonical pairs i.e. C-U instead of C-C (48,49). Our study confirmed this tendency. The G_2_C_4_ RNA formed slippery duplexes with two 3′ overhanging cytosines. In contrast to other studies, the terminal cytosines were ordered, and some were also engaged in the formation of a triplex structure.

### Pseudocanonical ANP77–cytosine pairs

In the G_2_C_4_–ANP77 structure, one ligand molecule was present. It interacted with the two overhanging cytosines of chain A, forming two pseudo-canonical base pairs (Figure 1D and H). The NUs of ANP77 mimicked the nitrogen bases of nucleotides, forming three H-bonds with the functional groups of the overhanging cytosines from chain A at their Watson-Crick edges (Figure 1E and F). A hydrogen bond was observed between the 2-amino group of ANP77 and the O2 carbonyl atom of cytosine, a second bond between the N8 imino group of the ligand and the exo-amino group of C, and a third interaction between the N1 atom of naphthyridine and the N3 function of cytosine. This H-bonding implies the protonation of one of the nitrogen atoms. Despite the high quality of the electron density map, the localization of the protons within the two pseudo-pairs was ambiguous (Figure 1F). One proton was most likely located at one of the NUs because, at neutral pH, ANP77 is single-charged. The second proton can be attributed to either cytosine or second NU (Supplementary Figure S1). The second pair of overhanging cytosines from chain D was ordered and interacted with symmetry-related RNA and the 2-exo amino group of the ligand (Supplementary Figure S2A-B).

### Conformation of ANP77 ligand

In the presented model, ANP77 exhibited a sandwich-like conformation, with the NUs shifting in relation to each other, resulting in limited stacking interactions between the aromatic moieties of the ligand (Figure 1C and E). One of the NUs overlapped extensively with the neighbouring 1G from chain B (Figure 1H), while the second unit did not form stacking interactions, despite its close proximity to the G–C pair of symmetry-related RNA molecules.

The aminopropyl carboxamide side chain of ANP77 was located in the major groove of RNA. Its amino-terminal region was disordered, indicating conformational freedom. Only the carbonyl group of the amide bond was modeled; however, it was in a double conformation. Depending on the position of the carbonyl group, it interacted with water molecules from the Hoogsteen edge of 5C involved in pseudo-pairing or with water molecules bridging the Hoogsteen edge of 4C from chain A (Figure 1F).

The crystallographic model of the G_2_C_4_-ANP77 complex confirmed that the ligand was capable of interacting directly with two consecutive cytosines. Through the formation of pseudo-base pairs, it merged into an RNA helix, extending the length of the double-stranded region (Figure 1H). This indicates that ANP77 fits into the structure not only in terms of H-bonding, but also in terms of the shape of the RNA helix. The geometry of the linker allowed the positioning of its aromatic rings in a similar manner as the nitrogen bases arranged in a nucleic acid helix. The NUs stacked one above another and were approximately 3Å apart, reflecting the value of the rise parameter in A-RNA form. Moreover, they were twisted to each other by approximately 25°, and the degree of twist could easily be adjusted by rotation around the C11-C12 bond (Figure 1C). The length of the propane linker seemed to be properly selected, as it allowed the binding of only two consecutive cytosines. Adding more carbon atoms would increase the flexibility and conformational freedom of the ligand, which could have a negative impact on ANP77 selectivity.

In light of structural data, ANP77 seemed to be a well-designed small molecule for sensing single stranded neighbouring cytosine residues in RNA. In practice, the ANP77 could be considered as an independent ligand molecule targeting C-rich sequences in RNA or as a part of a larger multivalent compound (50). In the single or multimeric form, ANP77 could bind to the G_2_C_4_ repeats and block sequestration of proteins, preventing formation of nuclear foci. Alternatively, ANP77 could be attached to the other heterocycle locally activating target RNA degradation by RNase L (51). Another application could explore fluorescent properties of ANP77 *in vivo* as an indicator of the Y/CC motifs in the RNA structures as it was shown earlier (37).

Using ANP77 as a tool in medicine raises the question about possible off-target effects since the Y/CC motif can be present in different RNA molecules. However, the previous research using SELEX and UV-melting method showed that the ligand requires not only the presence of two consecutive cytosine residues in single stranded region but also the neighbouring sequences play important role in RNA-ligand interaction (26,36,37). Moreover, crystallographic data indicated that the structural arrangement of cytosine residues is important for ANP77 recognition. Upon ligand binding the cytosines had to be in a single stranded form but in a way to maintain helical character of double-stranded region. In case of the G_2_C_4_ repeats the off-target effect could be also minimized by the high abundance of mutated transcripts in relation to other RNA molecules. Nevertheless, the further improvement of selectivity against specific RNA could be obtained by modification of the ANP77 molecule or incorporation into the larger, multivalent compound.

### How to improve the properties of the ANP77 ligand

The ANP77 molecule was designed to interact with two adjacent cytosines. Therefore, a number of features had to be taken into consideration during development of the ligand to ensure specific interaction with the target sequence (26,36). First, functional groups of NUs were selected as an interface for direct interactions with Watson–Crick edges of cytosines. Second, the aromatic rings were connected by short linker consisting of three carbon atoms in order to restrict conformational freedom of the molecule and facilitate formation of the stacked structure of the ligand. Third, the ANP77 possessed aminopropyl carboxamide side chain which easily undergo protonation increasing water solubility and interactions with negatively charged nucleic acids.

Our model provided the opportunity to rationally design modifications of ANP77 to improve its interaction with RNA in terms of the specificity, stability, and structural features of the target sequence. One modification would be to induce the protonation of both NUs because, at a neutral pH, only one of the nitrogen atoms (N1 or N1’) possesses proton (36). In the crystal structure, it acted as a hydrogen-bond donor for cytosine's N3 imino group. In turn, a maximum of three H-bonds were formed between naphthyridine and cytosine. Securing the protonation of both units could facilitate tight binding and increase the ligand affinity for the target RNA. The second modification could involve redesigning the side chain of the ligand, which is located close to the major groove of the helix. The electrostatic potential generated by exo-amino groups of cytosines was predominantly positive, and most of the exo-amino groups interacted with water molecules, indicating their binding potential. Replacing the protonated amine located at the end of the side chain with a more negatively charged group could be more beneficial. One possible example is the hydroxyl group, which has an electronegative character and can serve as an acceptor of the H-bond.

### Unliganded structure of G_2_C_4_ RNA

**Figure 2.**
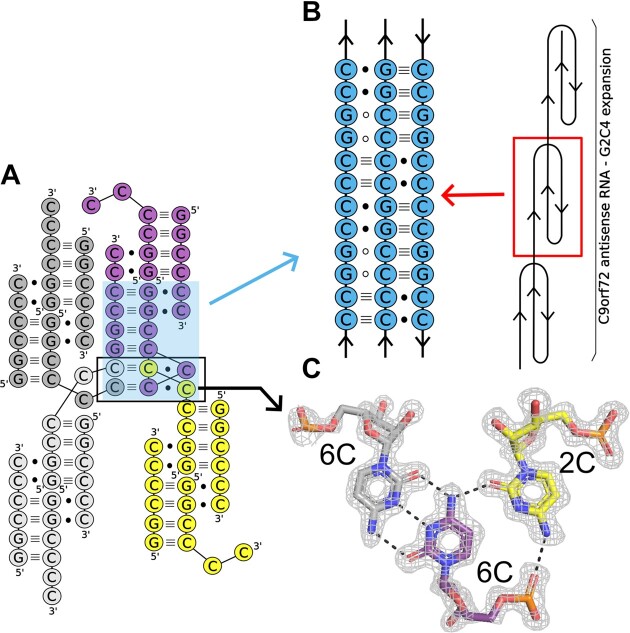
Higher-order interactions of unliganded RNA containing G_2_C_4_ repeats. (**A**) Cytosine residues of three symmetry-related molecules (grey, yellow and purple) form two unique C•C^+^•C base triples (black box). (**B**) Proposed model of triplex structure formed by expanded G_2_C_4_ repeats (see text for details). Open circles denote close proximity of G residues and/or H-bond interactions with the C-G pairs. (**C**) Example of the C•C^+^•C triple assembled by the trans C•C^+^ pair (purple and gray) found in the i-motif structure and cytosine (yellow) located in the Hoogsteen edge moiety. The 2Fo–Fc electron density map (grey) is contoured at the 1σ level. The H-bonds are represented by black dashed lines.

We determined the crystal structure of G_2_C_4_ in its free form at pH 6.0 (Protein Data Bank code: 8QMI) and at pH 6.5 (Protein Data Bank code 9EN6) (Supplementary Table S1). The superposition of both structures showed high similarity with r.m.s.d. of 0.38 Å. The overall assembly of the triplex structure remained the same. The two terminal cytosines of chain A, which were bound to ANP77 in the liganded structure, engaged in higher-order interactions (Figure 2A). They formed peculiar C•C^+^•C base triples assembled by symmetry-related residues (5C of chain A, 6C of symmetry-related chain A, and 6C of symmetry-related chain D) (Figures 2A and C). The base triples consisted of a trans C•C^+^ pair commonly found in i-motif structures. The N-glycosidic bonds were oriented in trans relative to each other and one of the cytosines (the N3 atom) was protonated. The C•C^+^ pair formed three hydrogen bonds *via* Watson–Crick edges. The third C from the triples was located in the Hoogsteen edge of one of the cytosine residues from the i-motif pair. It interacted with two H-bonds between the wedged cytosine and the cytosine from the C•C^+^ pair (Figure 2C).

The unique triplex-i-motif observed in ligand-free G_2_C_4_ RNA has not yet been described. This emphasizes the potential of cytosines to assist in the formation of higher-order RNA structures, presenting an example of diverse RNA folding pathways. In our opinion, the structural richness of G_2_C_4_ RNA could be implemented in structural biology, that is, in the folding of RNA nanoparticles, the assembly of RNA into multimers for CryoEM measurements, or the enhancement of crystal lattice formation during crystallization. In the context of expanded G_2_C_4_ repeats, the obtained results indicated that the G_2_C_4_ RNA antisense strand can fold not only into simple hairpins, but also into diverse three-dimensional structures (triplexes or i-motifs). Based on our data, we propose a model in which the long tracts of G_2_C_4_ repeats form long triplexes consisting of three different base triples: C•C^+^•C, C^+^•G–C, and G•C–G (Figure 2B). This structural arrangement allows cytosines to engage in more favorable H-bond interactions than those observed in the crystal structure of the G_2_C_4_ duplex. Triplexes are involved in the folding of RNAs into complex three-dimensional architectures, and they are crucial for the biological activity of RNA, such as telomere synthesis, ribosomal frame-shifting, regulation of gene expression through metabolite sensing, and the protection of RNA from degradation (52–61). The triple RNA helix formed by G_2_C_4_ repeats can be another example of a triplex that plays an important role in cells, exemplifying the pathological role of HRs. In that case the triplex of RNA G_2_C_4_ repeats, next to hairpin, tetraplex or i-motif structures, could be also involved in protein sequestration and nuclear foci formation or serving as a binding site of ribosomes during RAN-translation process. In case of the physiological range of HRs the triplex structure could stabilize the mRNA or participate in binding of specific regulatory factors.

### Interactions between RNA molecules and ions

In both structures interactions between RNA and ion species were observed. In the liganded structure three chloride ions were located: two in the major groove of the triplex motif and the third ion between the end of the triplex and beginning of the flanking duplex (Supplementary Figure S3A). All ions interacted with the exo-amino groups of cytosine residues. The distance between acceptor and H-bond donor was within the range of 3.2–3.4 Å, confirming the appropriate interpretation of electron density peaks. In the unliganded structure two magnesium cations were observed. Both of them directly interacted with oxygen atoms of the phosphate groups of RNA (Supplementary Figure S3B). The first one was bound to the 4C residue of chain A. Its coordination sphere consisted of the oxygen atom and five water molecules, typical for magnesium cations. The distances between acceptors and donors were in the range 2.0–2.3 Å. One of the water molecules from the magnesium coordination sphere interacted also with the oxygen atom of the phosphate group of the symmetry-related 6C residue of chain B (Supplementary Figure S3B). The second magnesium ion was located on a 2-fold axis between two phosphate groups of symmetry-related RNA molecules. Simultaneously, cation was directly bonded to two oxygen atoms (one O atom from phosphate of the 6C residue of chain C and second O atom of symmetry-related 6C residue of chain C) and four water molecules. The H-bond distances were also within the 2.0–2.2 Å range.

The interactions between RNA molecules and cations can provide information about local charge generated by the functional groups of the nucleotides. In case of the G_2_C_4_ sequence the abundance of cytosine residues resulted in the positive charge of the major groove which was attracted by the chloride anions. The similar effect was observed for oligomers containing the UGG motif (62). The major groove of the helix was negatively charged by carbonyl groups of guanosine residues. This attracted cations, especially Ba^2+^, whose presence increased thermal stability of RNA duplexes. For the phosphate groups, being in close proximity, their high negative charge was usually neutralized by Mg^2+^ ions as it was observed in unliganded structure. In this case the Mg^2+^ participated in the crystal lattice formation but in many RNA crystal structures it was shown to be crucial for proper RNA folding and activity (63).

### Biochemical analysis of G_2_C_4_–ANP77 complex

**Figure 3.**
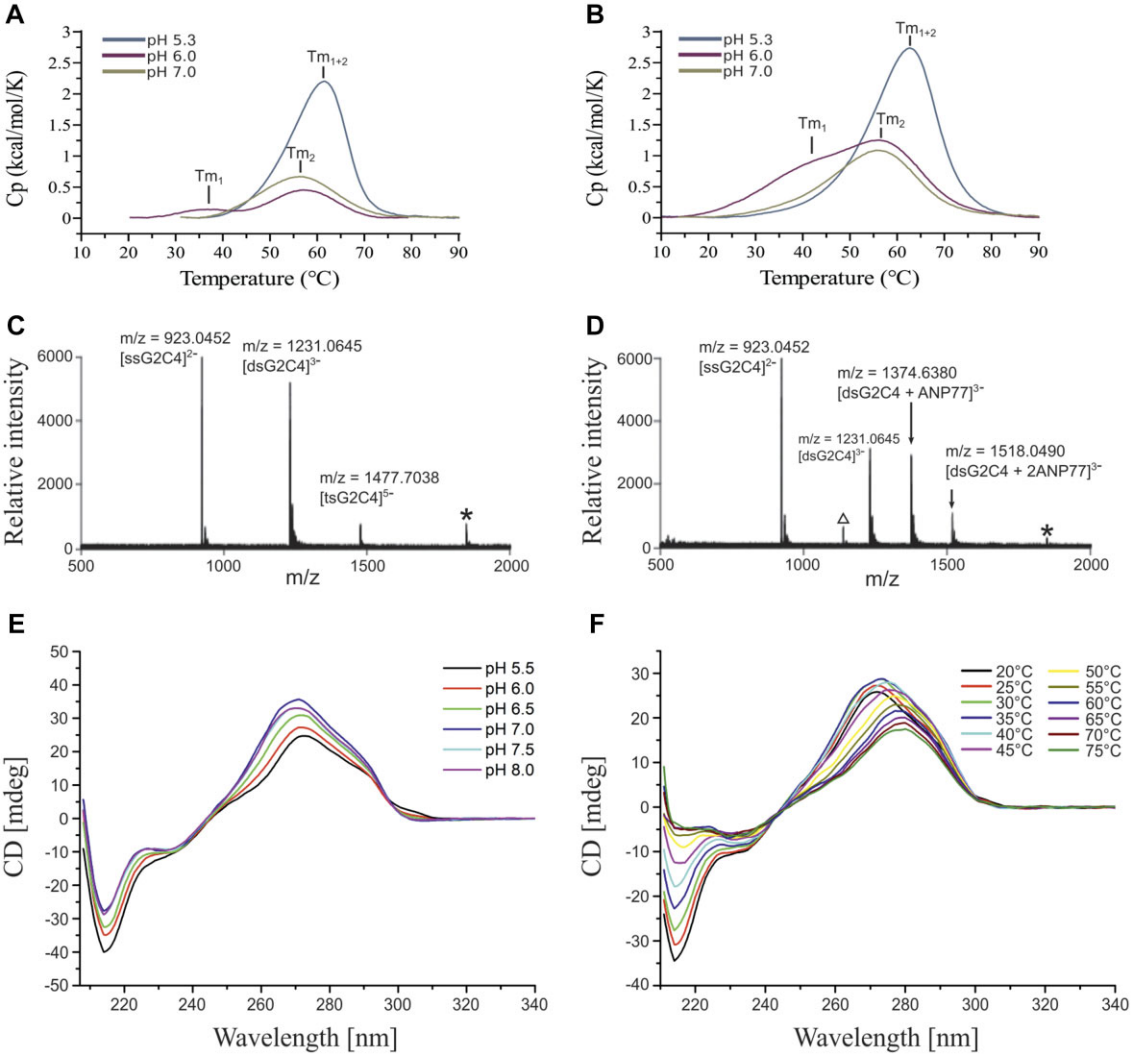
Bio-physical evaluation of interactions between RNA and ANP77 ligand. (A, B) Differential scanning calorimetry (DSC) spectra measured for (**A**) RNA oligomer and (**B**) for RNA-ligand complex in different pH conditions. (C-D) Cold-spray ionization time-of-flight mass spectrometry (CSI-TOF-MS) of G_2_C_4_ RNA (20 mM) in the (**C**) absence and (**D**) presence of ANP77 (50 mM). Asterisk (*) represents an ion corresponding to the 2– ion of dsG_2_C_4_ overlapping with the 1– ion of ssG_2_C_4_. The open triangle represents ion that could not be assigned. ssG_2_C_4_, dsG_2_C_4_, and tsG_2_C_4_ denote single-stranded, double-stranded, and tetra-stranded G_2_C_4_ RNA, respectively. (E, F) Circular dichroism spectra of G2C4 RNA (30 μM) measured in different pH (**E**) and temperatures (**F**).

Differential scanning calorimetry (DSC) was used to assess the thermodynamics of ligand binding and folding of G_2_C_4_ RNA. Similarly to crystallization experiments, we performed measurements at pH 6.0. Two peaks were observed for the unliganded RNA, indicating melting of the RNA structure: Tm1 (triplex melting point) at 38.1°C and Tm2 (duplex melting point) at 56.8°C (Figure 3A). In the presence of ANP77, Tm1 was shifted to 41.5°C, while Tm2 slightly changed to (57.4°C) (Figure 3B and Supplementary Table S2). Considering the importance of pH for the protonation of cytosines in terms of the folding of G_2_C_4_ RNA into a triplex structure and ligand binding, we performed DSC measurements at pH 7.0 and 5.3. At pH 7.0, for unliganded G_2_C_4_, only a single peak (Tm2) was detected at 55.3°C (1.5°C lower than Tm2 at pH 6.0) (Figure 3A). The addition of the ligand did not alter the DSC profile (single peak at 54.4°C) (Figure 3B and Supplementary Table S2). At pH 5.3, one peak Tm1 + 2 (merged melting points of triplex and duplex) was also detected, but at higher temperatures, either for RNA alone (Tm1 + 2 = 59.6°C) and the RNA-ligand complex (Tm1 + 2 = 60.2°C). However, the height and profile of the peak at pH 5.3 was different from those at pH 6.0 and 7.0.

Mass spectra indicated the presence of double- and tetra-stranded form of unliganded G_2_C_4_ RNA and only a double-stranded form of the RNA-ligand sample. In the latter, two complexes could be distinguished, representing 1:1 and 1:2 RNA-to-ligand ratios, which are consistent with the crystallographic model showing two potential ANP77 binding sites (Figure 3C and D).

CD measurements were performed for RNA composed of one, two, and three G_2_C_4_ repeats at six different pH values (from pH 5.5 to 8.0). The CD spectra of all oligomers at pH 5.5 showed a similar profile: the high negative peak at 215 nm and the high broad positive peak at 272 nm (Figure 3E and Supplementary Figure S4). At higher pH values we observed a decrease in the peak height at 215 nm and a shift of the positive peak from 272 nm to 270 nm (Figure 3E and Supplementary Figure S4). The most significant changes in CD spectra were detected between pH 5.5 and 6.0. The obtained CD spectra, together with the observed reduction of the negative peak at 215 nm and shift of positive peak at higher pH suggests the presence of triplex structure which is in agreement with previous studies (64–66). The CD method was also used to identify the structural changes upon melting of the G_2_C_4_ oligomer at pH 6.0. Measurements were performed from 20 to 75°C (5°C step). Two sequential transitions could be distinguished: one from 20° to 35°C and second from 40° to 75°C. In the first transition, the 215 nm negative peak increased from −34.49 to −22.76 mdeg while the 272 nm peak decreased only slightly (Figure 3F). In the second transition, the negative peak disappeared at 55°C, while the 272 nm peak decreased significantly and shifted to 280 nm. Finally, CD measurements were also performed for the RNA oligomers having two and five repeats in the presence and absence of ANP77 ligand. Binding of the ligand induced spectral changes in the range of 300–370 nm (Supplementary Figure S5) which may result from the fact that the achiral ANP77 became a part of the chiral RNA-ANP77 complex.

In order to obtain more information regarding the binding of ANP77 to investigated RNA we preformed ITC measurements for G_2_C_4_ RNA having one, two and three repeats. The G_2_C_4_ RNA oligomer interacted with ANP77 with *K*_d_ of 7.9 ± 1.0 μM (Supplementary Figure S6A). In case of (G_2_C_4_)_2_ the ITC showed the best fit to a sequential binding sites model assuming the presence of two binding sites (Supplementary Figure S6B). The average *K*_d_, obtained from two separate experiments, for the first binding site was 976 ± 377 nM while for the second binding site the *K*_d_ was 7.6 ± 4.2 μM. The data obtained for titration of the (G_2_C_4_)_3_ could not be fitted and were unrepeatable (data not shown).

The affinity of ANP77 obtained for G_2_C_4_ RNA was rather low. This may be due to a fact that in solution two overhanging cytosines most likely presented structural freedom influencing the interaction with ANP77. In case of the (G_2_C_4_)_2_ RNA two binding sites were identified with different affinity which suggested potential structural heterogeneity of the sample and coexistence of different structural folds of G_2_C_4_ RNA. The low affinity binding site (with similar *K*_d_ as for G_2_C_4_ RNA) may come from the triplex structure while high affinity binding site may represent single stranded cytosines located in constrained environment (inside the RNA duplex). The coexistence of multiple structural forms of longer G_2_C_4_ RNA has been observed by others (19). This potential heterogeneity could also explain why we were unable to obtain crystals of complexes having two and more G_2_C_4_ repeats.

The native PAGE electrophoresis of (G_2_C_4_)_1–3_ oligomers showed that all oligomers migrated slower than the reference markers of the same length, suggesting formation of inter-molecular structures (Supplementary Figure S7). For the (G_2_C_4_)_3_ oligomer additional fast migrating band was observed suggesting the presence of intra-molecular species.

### X-ray structure versus biochemical data

The results of the biophysical evaluation can be interpreted in terms of structural data. In G_2_C_4_ RNA, ANP77 interacted with cytosine residues located in the single-stranded region. Although six H-bonds were formed between RNA and the ligand, the stacking interactions were limited, and the entropy of the cytosines was reduced, resulting in an elusive thermal effect. DSC measurements, CD and mass spectra indicated that in solution, G_2_C_4_ existed as a tetramer folded into a triplex structure (Figure 3). The best conditions for triplex formation were slightly acidic (pH 5.5–6.0). At pH 6.0 the DSC and CD-melting spectra showed two peaks and two structural transitions, respectively (Figure 3A and F). The first peak and the first transition likely corresponded to the melting of the Hoogsteen interactions in the base triples, whereas the second peak and transition represented the melting of the slippery duplex. The DSC spectra at pH 5.3 also suggested the presence of one stable structure, either triplex or i-motif. Under acidic conditions the triplex motif could be stabilized, resulting in the merging of the duplex and tetramer signals into one peak (46). Alternatively, i-motif could be present since it can be easily formed at pH < 5.5, although the CD spectra did not support this hypothesis (67,68). At pH 7.0, the protonation of cytosines required for base triple formation was more difficult, explaining the sole presence of duplex species. Similar results of DSC and CD measurements were obtained by Dodd et al. (19). They investigated RNA composed of different numbers of G_2_C_4_ repeats. The authors suggested the presence of alternative structures including slipped intermolecular and intramolecular states or non-canonical structures.

pH-dependent stabilization of the pyrimidine major groove triplexes was demonstrated by thermodynamic and NMR studies. Under acidic conditions, a higher efficiency of cytosine residue protonation results in the tightening of Hoogsteen interactions in triple base pairs (65,69,70). However, this requirement for protonation to form tertiary contacts does not imply a low stability of this motif in the cellular environment (71). Although the calculated pKa of the isolated cytosine was below pH 5.0, the structural context and solvent content indicate the protonation of C, even under basic conditions (72,73). The p*K*_a_ shift in cytosine has been observed in several crystal structures of pseudoknot from Beet Western Yellow Virus, Pea Enation Mosaic Virus and HDV ribozyme (57,74–78). These studies demonstrated that formation of the C^+^GCA motif was crucial for proper folding and RNA activity. The protonated cytosine residue was engaged in formation of junction of two helical stems by forming H-bonds with Hoogsteen edges of G–C pair and neighboring adenosine residue. The assembly of G_2_C_4_ into the triple helix also requires the protonation of cytosines. Our crystallographic and biophysical results, together with previous reports showed that C^+^ species can be observed under near-physiological conditions, suggesting that they can also be stable in the cellular matrix and/or inside insoluble nuclear foci (18,79).

Structural polymorphisms of the sense and antisense transcripts of HR result in complex pathological pathways in ALS/FTD. Our study demonstrates the potential of C-rich G_2_C_4_ repeats to form higher-order structures. The ‘driving force’ for RNA folding are cytosine residues. The limited nucleotide composition of G_2_C_4_ RNA results in a p*K*_a_ shift of cytosines, extending their capabilities for H-bonding under near-physiological conditions. Consequently, a triplex structure, containing C^+^•G–C and unique C•C^+^•C i-motif base triples, is formed. In the cellular environment (particularly inside the nuclear foci) some local pH and ionic gradients as well as molecular crowding effects could stabilize HR repeats in the triplex form. Thus, the presented fold of G_2_C_4_ repeats is another example of RNA structural diversity and can be considered as a platform for RNA drug development against ALS/FTD.

ANP77 is a binding candidate for C-rich G_2_C_4_ RNA. It directly binds to adjacent cytosine residues and forms pseudo-canonical base pairs. This is our second study indicating that small molecules interact with the Watson–Crick edges of nucleotides. We previously showed that cyclic mismatch-binding ligand (CMBL) exhibited specificity toward adenosine residues in CAG repeats associated with polyglutamine disorders (33). We demonstrated that ANP77 and CMBL do not require the formation of ‘pockets’ for specific RNA recognition but rather the presence of particular structural motifs. Bioinformatic tools are one strategy for design and identification of lead compounds (24). The crystallographic data for ANP77 and CMBL ligands and its derivatives can be used for *in silico* predictions, providing detailed information about their structure and interactions with RNA which could accelerate drug development against RNA-driven disorders.

## Supplementary Material

gkae376_Supplemental_File

## Data Availability

Atomic coordinates and structure factors for the reported crystal structures have been deposited with the Protein Data Bank under accession numbers 8QMH and 8QMI. Diffraction images have been deposited in Macromolecular Xtallography Raw Data Repository at https://doi.org/10.18150/AJNJBE; https://doi.org/10.18150/FESTPM; https://doi:10.18150/FRXAN8.
