# Supplementary material for: Antisense RNA C9orf72 hexanucleotide repeat associated with amyotrophic lateral sclerosis and frontotemporal dementia forms a triplex-like structure and binds small synthetic ligand
Source: Nucleic Acids Res. 2024 May 13;52(11):6707–17. doi: 10.1093/nar/gkae376 (PMC11194091; doi:10.1093/nar/gkae376)
Supplement: gkae376_Supplemental_File [file gkae376_supplemental_file.pdf]

Antisense RNA C9orf72 Hexanucleotide Repeat Associated With*Amyotrophic Lateral Sclerosis* and Frontotemporal Dementia Forms A Triplex-Like Structure and Binds Small Synthetic Ligand

Leszek Błaszczyk[1]†, Marcin Ryczek[1]†, Bimolendu Das[2]†, Martyna Mateja-Pluta[1], Magdalena Bejger[1], Joanna Śliwiak[1], Kazuhiko Nakatani[2] and Agnieszka Kiliszek[1]*

[1] Institute of Bioorganic Chemistry, Polish Academy of Sciences, Z. Noskowskiego 12/14, 61-704, Poland

[2] Department of Regulatory Bioorganic Chemistry, SANKEN (The Institute of Scientific and Industrial Research), Osaka University, 8-1 Mihogaoka, Ibaraki, Osaka 567-0047, Japan

* To whom correspondence should be addressed, Email: kiliszek@ibch.poznan.pl

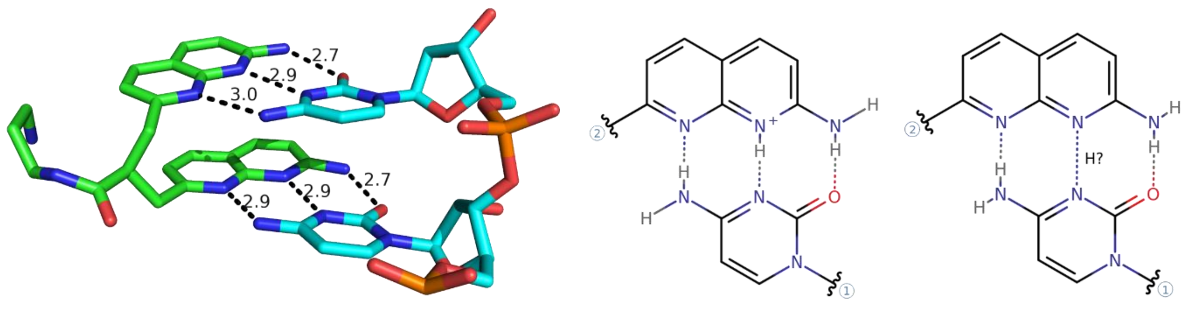

Supplementary Figure S1. The H-bond interactions between ANP77 (green) and cytosine residues (cyan) and potential localisation of protons in pseudo-canonical base pairs. See text for details.

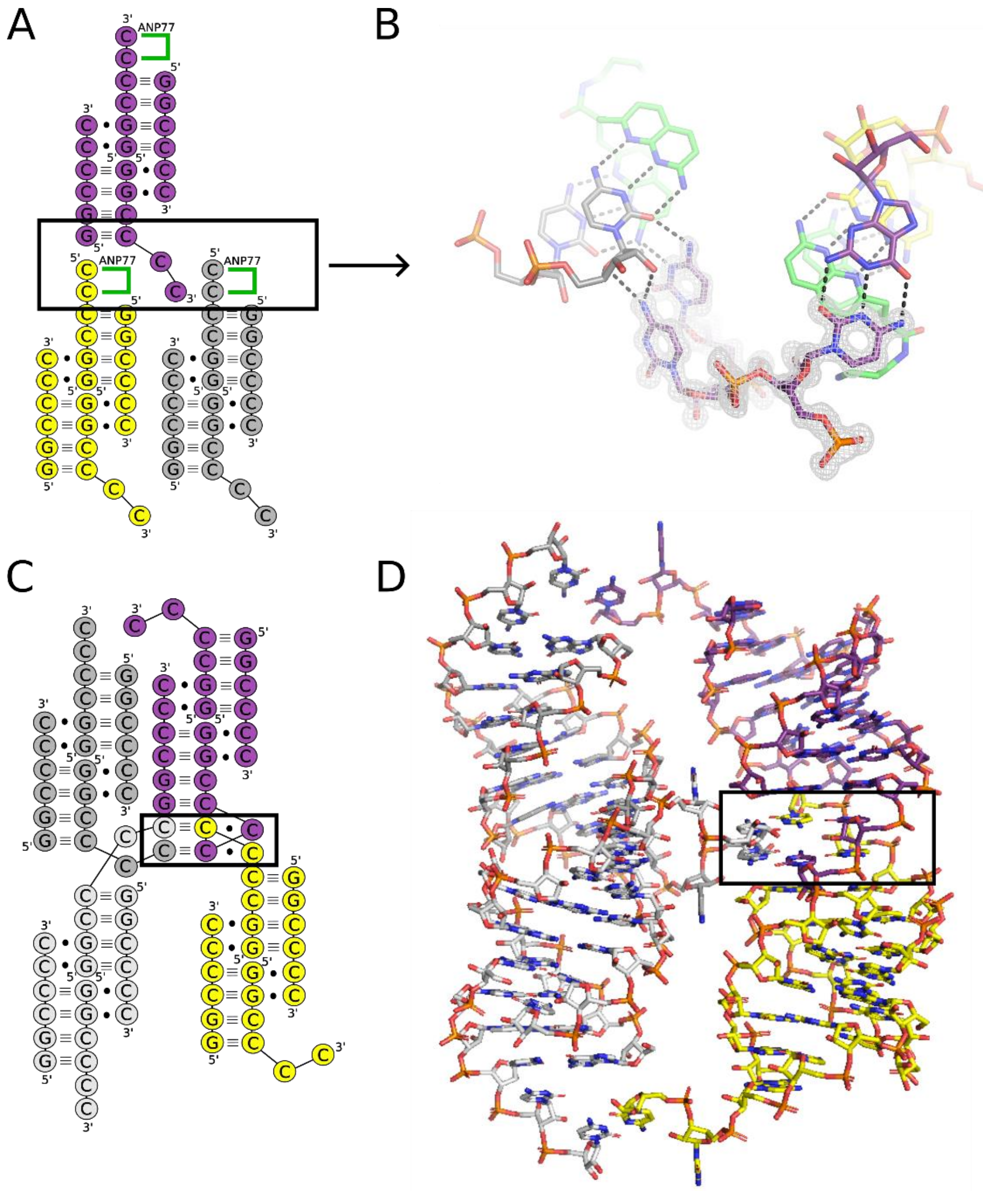

Supplementary Figure S2. Crystal contacts observed in the crystal lattice of RNA-ligand complex (A-B) and in unliganded model (C-D). (A and C) the schematic representation of crystal contacts between symmetry-related molecules of G_2C_4 RNA tetramers. In the liganded structure (A) the overhanging cytosines are located in the vicinity of pseudo-canonical base pairs formed between ANP77 and cytosines of symmetry-related RNA tetramer (black box). (B) Details of H-bond pattern observed between overhanging cytosines (purple sticks), cytosines (gray sticks) and ANP77 (green sticks) from symmetry-related molecule. (C and D) In the unliganded structure the respective cytosine residues are involved in formation of two unique base triples (black box).The 2Fo–Fc electron density map (gray) is contoured at the 1σ level. The H-bonds are denoted as black dashed lines.

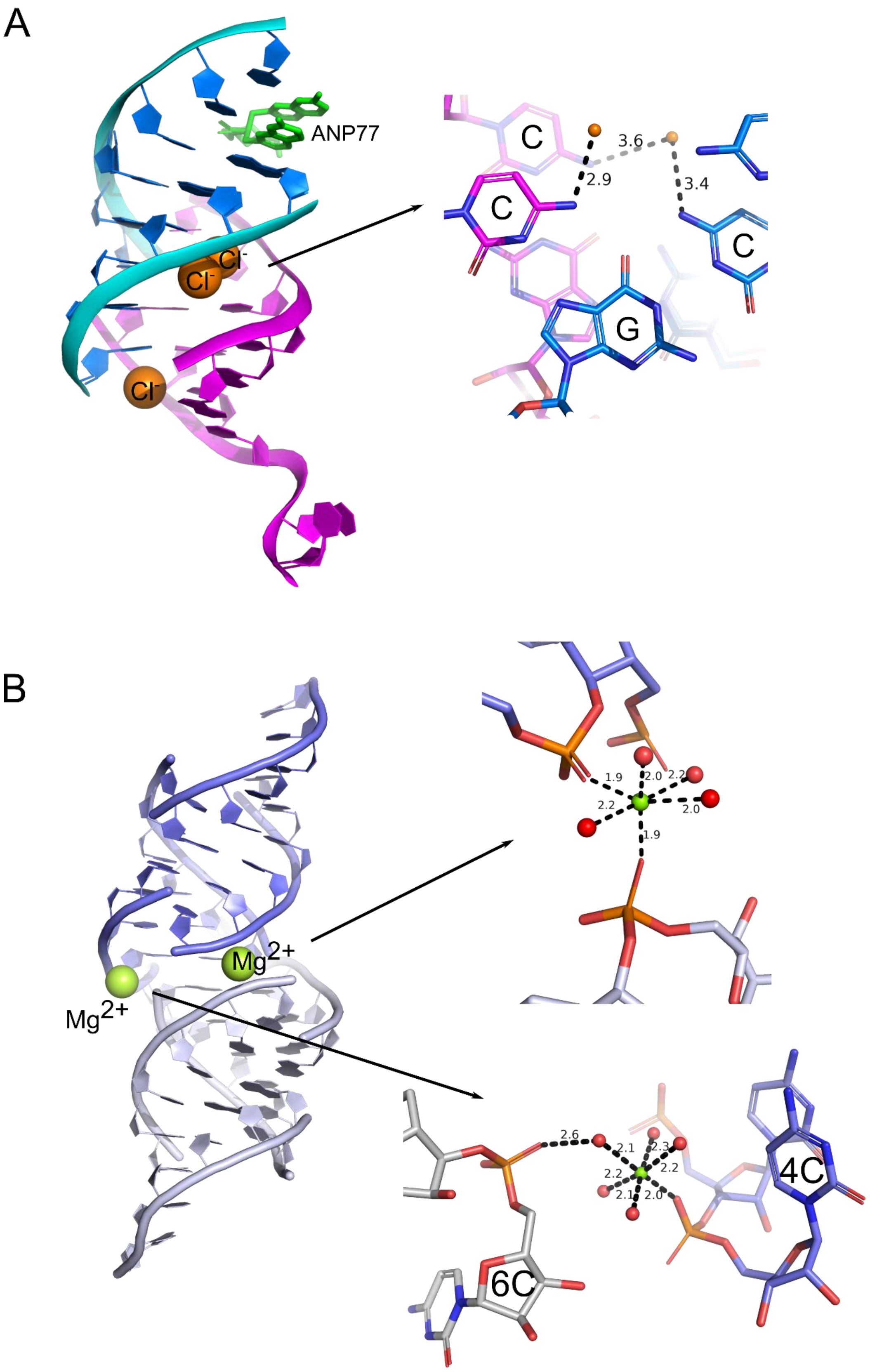

Supplementary Figure S3. The interactions between the RNA and ions in liganded (A) and unliganded (B) G2C4 structures. See text for details.

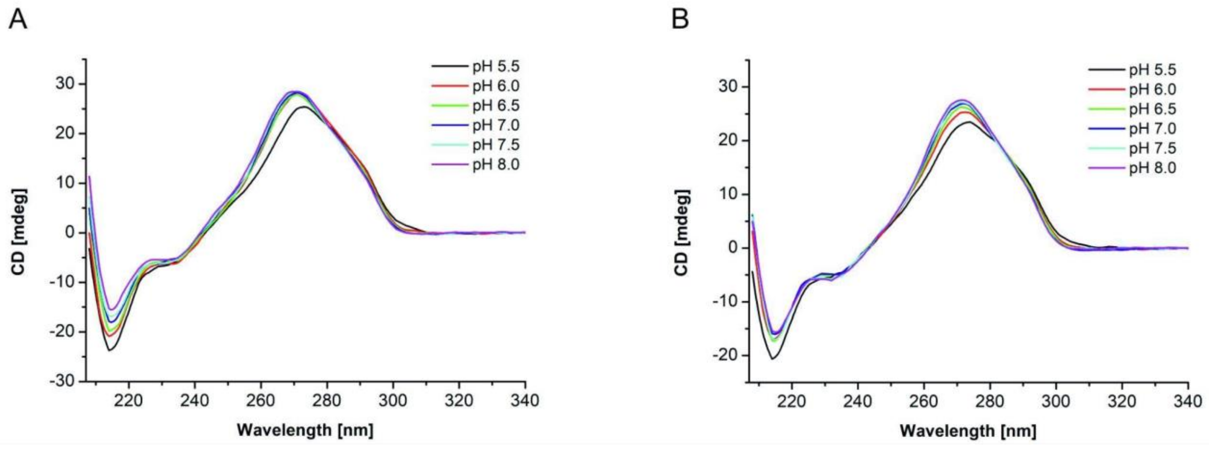

Supplementary Figure S4. Circular dichroism spectra measured in different pH for G2C4 RNA having two (A) and three (B) repeats.

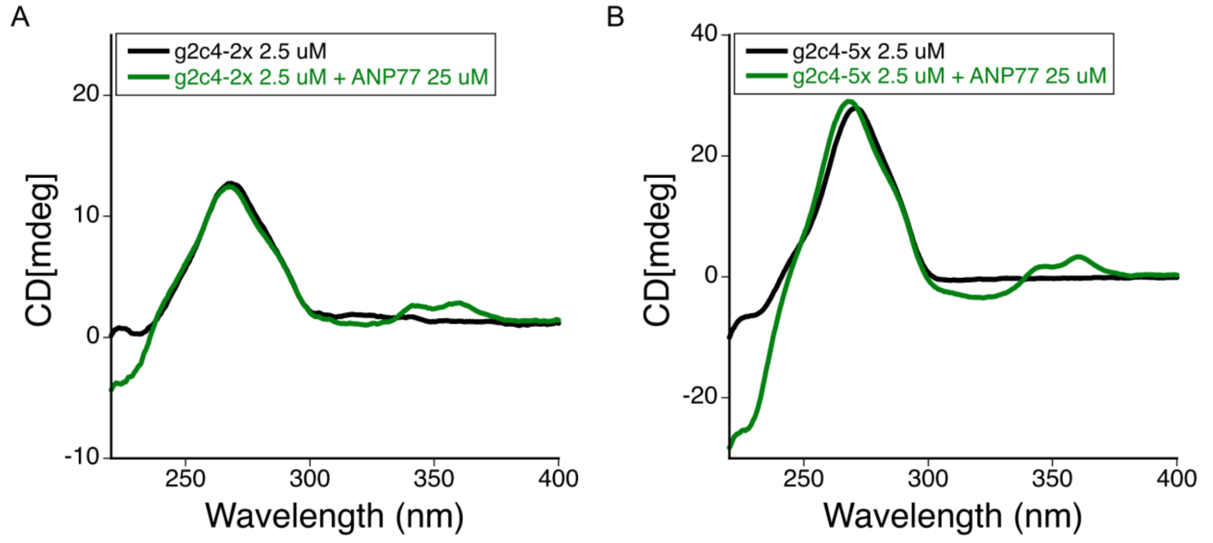

Supplementary Figure S5. CD spectra of G_2C_4 RNA having two (A) and five repeats (B) in the absence (black line) and presence (green line) of ANP77 ligand. Concentrations of RNA and ANP77 were 2.5 and 25 μM, respectively.

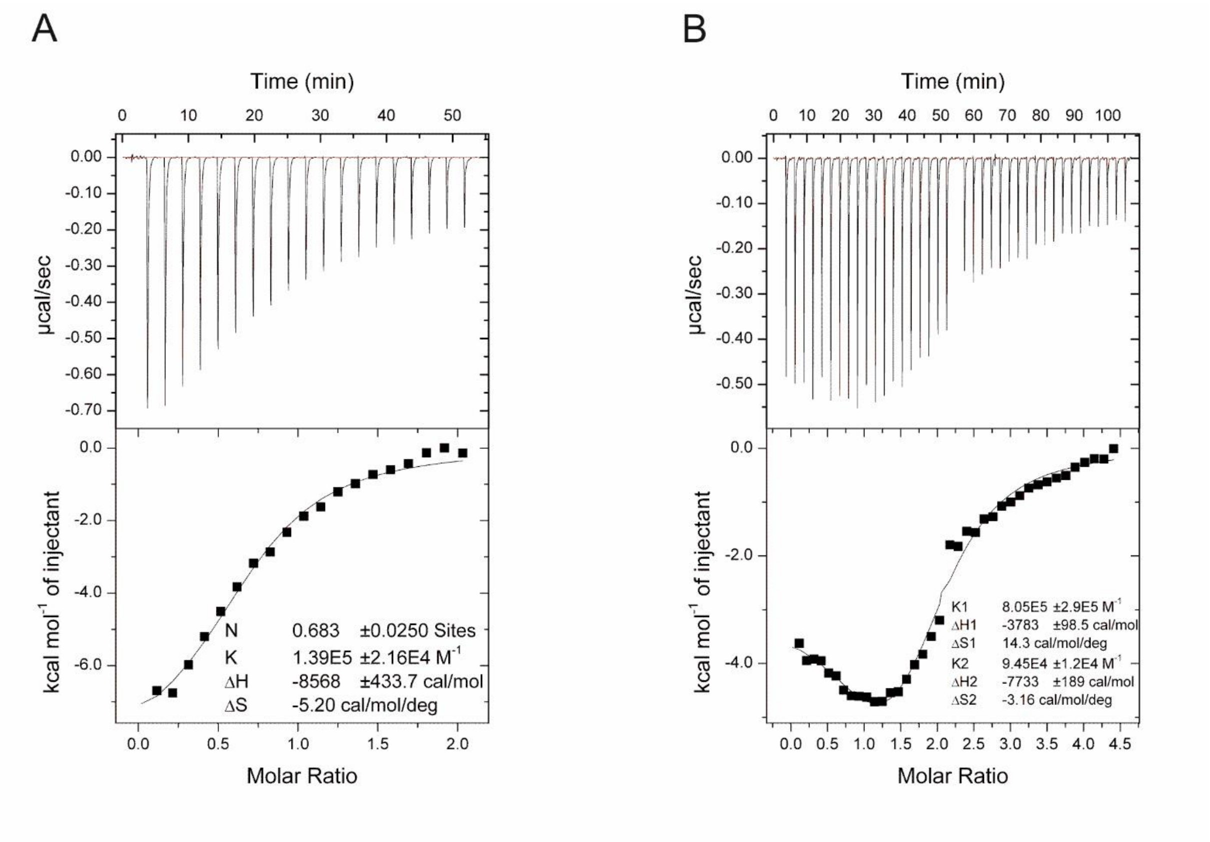

Supplementary Figure S6. Isothermal titration calorimetry of G_2C_4 (A) and $(G2C4)_2$ RNA (B) with ANP77 ligand. The top plot of each panel shows the raw heat data obtained from 19 (A) or 38 (B) consecutive injections of ligand into the sample cell containing RNA. At the bottom of each panel, the binding isotherm has been created by plotting the heat peak areas against the molar ratio of ANP77 to RNA present in the cell. The line represents the best fit of a model with one set of binding sites (A) or two sequential binding sites (B).

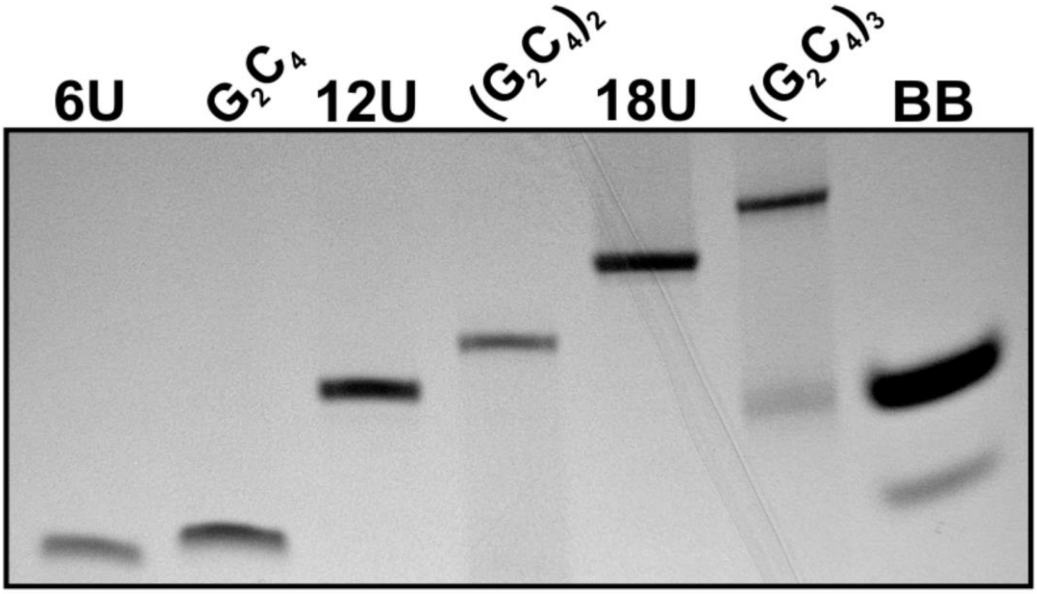

Supplementary Figure S7. Native PAGE electrophoresis of RNA oligomers containing one, two and three G2C4 repeats. 6U, 12U and 18U denote RNA oligomers composed of uridines that were used as markers. BB - bromophenol blue.

Supplementary Table S1. Summary of X-ray data and model refinement statistics.

	G_2C_4 + ANP77	G_2C_4 native	G_2C_4 native
**Data Collection**			
X-ray source	XtaLAB Synergy-R, HyPix	XtaLAB Synergy-R, HyPix	Dectris EIGER1 Si 16M,
Space group	$C222_1$	$C222_1$	$C222_1$
Cell parameters (Å)	a = 29.34, b = 45.18, c = 86.28	a = 41.76, b = 46.64, c = 51.49	a = 41.66, b = 45.84, c = 51.48
Resolution (Å)	21.85-1.10 (1.16-1.1)[a]	21.25-1.50 (1.58-1.50)#	25.75-0.92 (0.97-0.92)
R_{merge}	0.076 (0.560)	0.116 (0.553)	0.072 (1.347)
I/σ	26.9 (4.4)	10.0 (2.4)	17.2 (1.59)
$CC_{1/2}$	0.99 (0.89)	0.99 (0.86)	0.99 (0.73)
Completeness (%)	99.9 (99.4)	99.7 (100)	99.9 (99.8)
Redundancy	16 (11.0)	7.3 (4.8)	13.3 (12.8)
Number of unique reflections	23771	8334	34769
**Refinement**			
Software	Refmac 5.8.0405/ Phenix (1.20.1)		Refmac 5.8.0258
Number of reflections: work/test	22774/479	7905/404	33040/1726
Overall mean B value (Å^2)	13.03	8.62	12.536
R_{work}/R_{free}	0.1218/0.1737	0.1680/0.2254	0.119/0.132
RNA atoms	724	555	616
Water molecules	108	108	140
Ligand molecules	1 ANP77	2 Mg^{2+} ions	3 Mg^{2+} ions
RMSD in bonds (Å)	0.008	0.007	0.019
RMSD in angles (°)	1.4	1.9	2.7
PDB code	8QMH	8QMI	9EN6
x-ray images	doi:10.18150/AJNJBE	doi:10.18150/FESTPM	doi:10.18150/FRXAN8

[a]Values in parentheses are for the last resolution shell

Supplementary Table S2. Summary of Tm melting temperature measured by DSC in different pH.

	Tm_1 (°C)	Tm_2 (°C)	Tm_{1+2} (°C)
pH 5.3 native			59.6 ± 0.9
pH 5.3 + ANP77			60.2 ± 0.9
pH 6.0 native	38.1 ± 0.4	56.8 ± 0.2	
pH 6.0 + ANP77	41.5 ± 0.6	57.4± 0.4	
pH 7.0 native		55.3 ± 0.4	
pH 7.0 + ANP77		54.4 ± 0.3	
